# Polydimethylsiloxane Surface Modification of Microfluidic Devices for Blood Plasma Separation

**DOI:** 10.3390/polym16101416

**Published:** 2024-05-16

**Authors:** Margarida Gonçalves, Inês Maia Gonçalves, Joel Borges, Vera Faustino, Delfim Soares, Filipe Vaz, Graça Minas, Rui Lima, Diana Pinho

**Affiliations:** 1 Microelectromechanical Systems Research Unit, CMEMS-UMinho, University of Minho, Campus de Azurém, 4800-058 Guimarães, Portugal; b13836@cmems.uminho.pt (M.G.); vera.faustino@cmems.uminho.pt (V.F.); dsoares@dem.uminho.pt (D.S.);; 2 LABBELS—Associate Laboratory, 4800-122 Braga, Portugal, and 4800-058 Guimarães, Portugal; 3 MEtRICs, Mechanical Engineering Department, University of Minho, Campus de Azurém, 4800-058 Guimarães, Portugal; id9385@alunos.uminho.pt (I.M.G.); rl@dem.uminho.pt (R.L.); 4 IN+, Instituto Superior Técnico, Universidade de Lisboa, 1649-001 Lisboa, Portugal; 5 Department of Diagnostic and Therapeutic Systems Engineering, Institute of Biomaterials and Bioengineering, Tokyo Medical and Dental University, 2-3-10 Kanda-Surugadai, Chiyoda-ku, Tokyo 101-0062, Japan; 6 Physics Center of Minho and Porto Universities (CF-UM-UP), University of Minho, Campus de Azurém, 4800-058 Guimarães, Portugal; joelborges@fisica.uminho.pt (J.B.); fvaz@fisica.uminho.pt (F.V.); 7 LaPMET, University of Minho, 4710-057 Braga, Portugal; 8 CEFT, Faculty of Engineering, University of Porto, Rua Dr. Roberto Frias, 4200-465 Porto, Portugal; 9 ALiCE, Faculty of Engineering, University of Porto, Rua Dr. Roberto Frias, 4200-465 Porto, Portugal

**Keywords:** PDMS, microfluidic devices, surface modification, capillary studies, blood flow studies

## Abstract

Over the last decade, researchers have developed a variety of new analytical and clinical diagnostic devices. These devices are predominantly based on microfluidic technologies, where biological samples can be processed and manipulated for the collection and detection of important biomolecules. Polydimethylsiloxane (PDMS) is the most commonly used material in the fabrication of these microfluidic devices. However, it has a hydrophobic nature (contact angle with water of 110°), leading to poor wetting behavior and issues related to the mixing of fluids, difficulties in obtaining uniform coatings, and reduced efficiency in processes such as plasma separation and molecule detection (protein adsorption). This work aimed to consider the fabrication aspects of PDMS microfluidic devices for biological applications, such as surface modification methods. Therefore, we studied and characterized two methods for obtaining hydrophilic PDMS surfaces: surface modification by bulk mixture and the surface immersion method. To modify the PDMS surface properties, three different surfactants were used in both methods (Pluronic^®^ F127, polyethylene glycol (PEG), and polyethylene oxide (PEO)) at different percentages. Water contact angle (WCA) measurements were performed to evaluate the surface wettability. Additionally, capillary flow studies were performed with microchannel molds, which were produced using stereolithography combined with PDMS double casting and replica molding procedures. A PDMS microfluidic device for blood plasma separation was also fabricated by soft lithography with PDMS modified by PEO surfactant at 2.5% (*v*/*v*), which proved to be the best method for making the PDMS hydrophilic, as the WCA was lower than 50° for several days without compromising the PDMS’s optical properties. Thus, this study indicates that PDMS surface modification shows great potential for enhancing blood plasma separation efficiency in microfluidic devices, as it facilitates fluid flow, reduces cell aggregations and the trapping of air bubbles, and achieves higher levels of sample purity.

## 1. Introduction

The era of microfluidics started in the 1980s with the development of silicon etching procedures which were made for the microelectronics industry [1]. Throughout the years of developing microfluidic devices, microfluidic technology has achieved great technological advances, both for science and industry [2,3]. Microfluidics revolves around fluid behavior and its control, defined by dimensions that are on sub-millimeter and micrometer scales and integrated with micro- and nano-structures [4]. Micro- and nanofabricated devices have revolutionized our ability to manipulate small fluid volumes and particles, finding various applications such as chemical and biological characterization, sensors, cell counting and capture, micropumps and actuators, and more [5]. These advancements have brought engineering tools to a scale that matches the dimensions of the objects they handle [5].

Microfluidic devices changed as new fabrication technologies became available. The requirements, such as optical transparency and low-cost micrometer-sized channels, meant that materials such as glass and silicon were superseded by polymers and elastomers [6]. Polydimethylsiloxane, commonly referred to as PDMS, is a mineral–organic polymer composed of carbon and silicon that is widely used as a structural material for microfluidic devices and biochips for applications in life sciences, because of its ease of fabrication, inertness, and biocompatibility [7,8]. PDMS belongs to the siloxane family, and derives its functional group designation from silicon, oxygen, and alkane, where the Si–O bond gives the polymer its thermal and chemical stability [9]. PDMS is a hydrophobic material, exhibiting a water contact angle (WCA) of 110° [10]. However, in microfluidic devices used for biomedical applications, inadequate surface wetting leads to the inefficient flow of fluids through channels, causing flow instabilities, the trapping of air bubbles, and the non-uniform distribution of samples or reagents. In cell culture applications, the hydrophobic nature of PDMS surfaces can affect cell adhesion and spreading, leading to suboptimal cell growth and viability. Furthermore, the hydrophobicity of PDMS surfaces can also lead to the nonspecific adsorption of biomolecules, such as proteins, which can interfere with analytical assays or biological experiments. This adsorption can affect the accuracy and reliability of results, particularly in applications where precise control over surface interactions is crucial, such as biosensing and drug delivery systems. Overall, while the hydrophobic nature of PDMS offers advantages in certain applications, such as easy release of molded structures and compatibility with hydrophobic molecules, it can pose challenges in applications where interactions with aqueous solutions or biological fluids are essential. Addressing these challenges often requires surface modification techniques to enhance the hydrophilicity of PDMS surfaces and improve their performance in specific applications [10]. Therefore, modifications to the surface of PDMS that result in properties such as hydrophilicity, electrical conductivity and anti-fouling characteristics are of significant interest. In particular, for bioengineering applications, hydrophilic microchannels demonstrate the ability to increase cell adhesion, reduce absorption, and reduce air bubble trapping during the filling process with aqueous solutions [8,10]. The wettability of biomaterials is an essential property for ensuring the desired biological response, and its measurement represents an essential scientific evaluation of the properties of biomaterials. The most commonly used techniques to quantify the wettability of polymeric biomaterials surfaces are WCA measurements [11] and capillary flow studies [12].

In the domain of applications of biomicrofluidic devices, the separation of plasma from blood is central for blood disease diagnostics and prognosis [3,13,14,15] as it enables the isolation of key components from blood that can correlate to a specific pathological condition. Such devices are capable of presenting a detailed picture of the physiological condition of the human body due to a myriad of biomarkers that are found in human blood plasma, with the most common being proteins [16,17], electrolytes [18], urea, and glucose [19]. Thus, the study of PDMS surface modification to enhance hydrophilicity [20,21] holds great significance for the operation of passive microfluidic devices for blood sample preparation and plasma separation. The enhancement of hydrophilic properties facilitates the capillary movement of fluids and reduces cell wall adhesion and agglomeration, particularly of red blood cells (RBCs). The main purpose of this work was the fabrication of a microfluidic device for passive blood plasma separation [2,3], by using PDMS samples with improved wettability to assess plasma separation performance. PDMS surface modifications were performed by using different surfactants such as PEO, Pluronic^®^ F127 and PEG, and two methods for surface modification: bulk modification and surface immersion. The wettability of the modified PDMS samples was characterized by WCA measurements and capillary flow studies.

## 2. Materials and Methods

### 2.1. PDMS Samples Preparation

PDMS (Sylgard^TM^ 184, Ellsworth Adhesives Ibérica, Madrid, Spain) was prepared by mixing a base and a curing agent in mixing ratios of 10:1 and 5:1 (*w*/*w*). Both mixtures were blended with a spatula by mechanical force until a whitish color was obtained. The PDMS mixture was then degassed in a vacuum pump and poured into a Petri dish to be cured in an oven at 80 °C for 1 h. Cured PDMS samples were cut into rectangular blocks of 3 × 2.5 cm (length × width) with a thickness of 5 mm. These samples containing only a mixture of base and curing agent were considered the control PDMS samples.

#### 2.1.1. Bulk PDMS Modification Method

**Figure 1:**
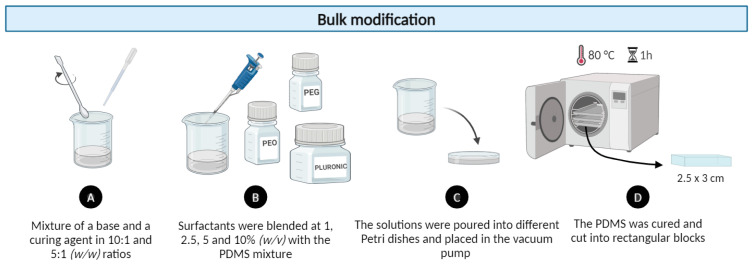
Experimental procedure of PDMS samples with bulk modification using 1, 2.5, 5, and 10% (*w*/*v*) of non-ionic surfactants: PEO, Pluronic^®^ F127, and PEG.

The bulk modification method refers to a process where the selected surfactants are blended with a PDMS mixture while all these components are still in a liquid form (see Figure 1). PEO, (C_2_H_4_O)_n_ [22], is an additive surfactant commonly used as a wetting agent for surface modifications due to its hydrophilic properties [23]. It is available in a very wide range of molecular weights, from 200 to 7.0 × 10^6^ g/mol, and low-molecular-weight PEOs are called PEGs [24]. Additionally, PEG derivatives have been extensively employed to modify PDMS and other hydrophilic materials to improve their biocompatibility [25]. Another surfactant that is widely used in numerous biotechnological applications is Pluronic, which belongs to a group of triblock copolymers with the general composition of (PEO)_m_(PPO)_n_(PEO)_m_. This copolymer has been proven to exhibit extremely low toxicity and immunogenic responses [26]. Furthermore, PEG- or PEO-modified surfaces have demonstrated a superior ability to resist protein adsorption [26]. For bulk modification, the surfactants PEO (Polysciences Europe GmbH, Hirschberg an der Bergstraße, Germany), Pluronic^®^ F127 (Sigma-Aldrich, Burlington, MA, United States), and PEG (Sigma-Aldrich, Burlington, MA, United States) were selected. The procedure for producing the PDMS mixture was repeated, at both 10:1 and 5:1 (*w*/*w*) ratios, and different percentages of surfactant (1, 2.5, 5, and 10% (*w*/*v*) were added based on the total volume of the PDMS mixture.

#### 2.1.2. Modification by Surface Immersion Method

**Figure 2:**
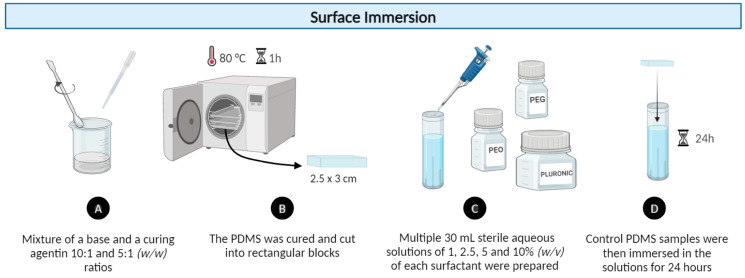
Experimental procedure for surface modification of PDMS samples by the immersion method with 1, 2.5, 5, and 10% (*w*/*v*) of non-ionic surfactants: PEO, Pluronic^®^ F127, and PEG.

In addition, a more direct approach of surface modification was adopted. The experimental process started with the PDMS sample fabrication described in Section 2.1. Subsequently, 30 mL of water solutions with 1, 2.5, 5, and 10% (*w*/*v*) of each surfactant (PEO, Pluronic^®^ F127, and PEG) were prepared. The PDMS samples, at 10:1 and 5:1 ratios, were immersed in the solutions with different surfactant percentages for 24 h at room temperature (see Figure 2). In this modification method, the surfactant molecules directly interact with the surface of the PDMS samples during complete immersion of the samples in the solution, resulting in intermolecular forces such as van der Waals interactions and hydrogen bonding between the PDMS surface and the surfactant molecules.

### 2.2. Contact Angle (CA) Measurements

Water contact angle (WCA) measurements were performed using an optical microscope connected to a computer with CA measurement software (SCA 20 1.0 software for measuring optical contact angle (OCA) and portable contact angle (PCA)) using the sessile drop method. Ten-milliliter drops of distilled water were dispensed with a micropipette onto the PDMS sample surface. In order to measure the WCA, the light, focus, baseline, and contour lines were adjusted with the aid of the software. In total, 30 measurements were performed per sample. The WCA measurements were taken immediately after the samples were produced, at the time points of 0, 2, 4, 6, 24, 48, and 72 h; 1, 2, and 3 weeks; and 1, 2, and 3 months. This approach allowed for a thorough analysis of the changes in the hydrophobic properties of the PDMS over time.

### 2.3. Microchannel Fabrication for Capillary Studies

#### 2.3.1. Microchannel Design and 3D Printing of the Molds

**Figure 3:**
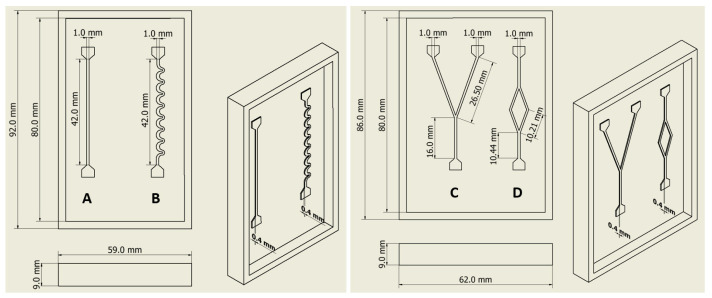
Designs and dimensions of the different molds for the capillary microchannels. Four channels were drawn: (A) a straight rectangular channel; (B) a channel with spiral-shaped geometry; (C) a channel with a main channel that bifurcates into two equal branch channels; and (D) a channel with bifurcation-confluence geometry.

The fabrication of the polymeric microfluidic devices for the capillary assays started with the drawing of a pattern design using the computer-aided design software Inventor (Inventor 27.0, student license). Four channels were drawn: (1) a straight rectangular channel with a length of 4.2 mm and width of 1 mm (Figure 3A); (2) a channel with spiral-shaped geometry with a length of 42 mm and width of 0.5 mm (Figure 3B); (3) a channel with a main channel with a width of 1 mm that bifurcates into two equal branch channels (Figure 3C); and (4) a channel with bifurcation-confluence geometry (Figure 3D). All channels had a depth of 0.4 mm. 

To obtain the microchannel molds, the drawings were transformed into .STL to be sent to the 3D printer pre-processing software, CHITUBOX V19.4 (Shenzhen, China, CBD-Tech). The printer used was the ELEGOO Mars (Shenzhen, China), a 3D stereolithography printer with integrated UV light. A specific translucent photosensitive resin (Standard LCD, Elegoo, Shenzhen, China) was used in the printing process and the high-resolution parameters were defined. Support structures and object orientation (45°) are essential to provide stability and resistance during the printing of each object layer and are defined as the standard of the printing. Post to the printing process, the remaining uncured resin was removed with isopropyl alcohol. Subsequently, the microchannel molds underwent a 10 min ultrasonic bath to remove any excess resin that remained on the microchannel surface mold. Afterward, it was subjected to a UV curing session lasting 2 h in the MERCURY Curing Machine (ELEGOO, Shenzhen, China). The UV light further cured the resin, making the molds more rigid, improving their structural integrity. The curing time may vary based on the resin type and the UV source’s intensity. Lastly, the supports were removed, and the mold was exposed to a 24 h thermal treatment at 80 °C in an oven. 

#### 2.3.2. PDMS Replica Molding and Double Casting Procedure

**Figure 4:**
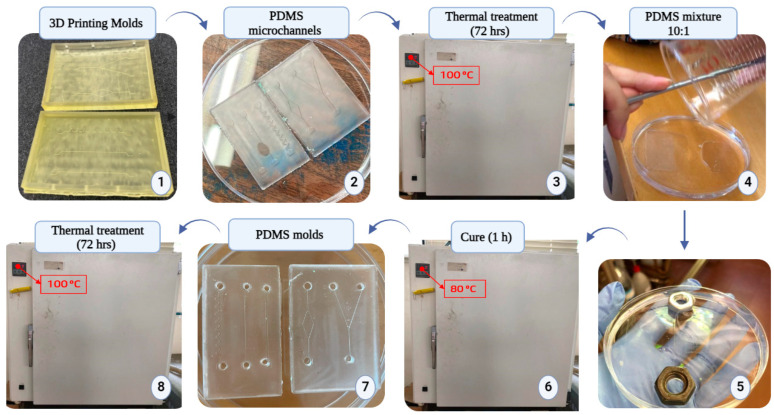
Steps of the double casting procedure. After making the PDMS molds using the 10:1 mixture (2), they were subjected to a 72 h heat treatment at 100 °C. Afterward, a new PDMS mixture was poured over the PDMS microchannels (4) (weights were employed to prevent the molds from floating) (5), and the replica mold procedure was performed followed by a thermal aging treatment. A new PDMS mold was obtained (7) and used for replica molding with modified PDMS.

After obtained the microchannel molds (Figure 4(1)), a procedure of replica molding was performed. Thus, a mixture of PDMS with a ratio of 10:1 was poured into the printed master molds. The molds containing the poured PDMS were degassed under a vacuum pump and cured in an oven (60 °C, 2 h). Once cured, the PDMS was peeled off and control PDMS replicas of the microchannels were obtained. 

Difficulties were encountered during the curing process when using bulk-modified PDMS, i.e., the modified PDMS in direct contact with the mold material did not solidify. Therefore, a double casting procedure [27,28] was implemented. Briefly, the obtained control PDMS channels were used as the molds (Figure 4(2)), but prior to being used, they were subjected to a thermal aging process [27] by spending 72 h in an oven at 100 °C (Figure 4(3)). Then, the thermal-treated control PDMS microchannels were placed in a Petri dish and filled with the 10:1 PDMS mixture (Figure 4(4,5)). A new degassing step was applied and the PDMS was cured at 80 °C for 1 h (Figure 4(6)). Subsequently, new PDMS molds were demolded from the PDMS microchannels (Figure 4(7)) and exposed to a thermal aging procedure (100 °C for 72 h; Figure 4(8)). Following this, new PDMS microchannel molds were obtained and could be used for the replica molding procedure with the bulk-modified PDMS mixtures. Figure 4 shows the double casting procedure and replica molding for the capillary microchannels.

Lastly, the fabricated channels were permanently sealed with a thin layer of PDMS with a 2 mm thickness. The thin layer was pre-cured at a temperature of 65 °C for a minimum time of 30 min. Subsequently, the clean microchannel replicas obtained from the PDMS mold were poured over the thin layer and bonded at 80 °C for 1 h in an oven. This resulted in sealed control and modified microchannels that were ready for capillary tests. Inlet and outlet ports were made using a biopsy punch with a diameter of 4 mm.

Note that the control PDMS samples and microchannels treated with surface activation using oxygen (O_2_) plasma were also obtained to compare our results with those in literature. The PDMS samples were positioned inside a vacuum chamber of a ZEPTO equipment set-up (Diener, Germany), with a base pressure of 20 Pa. O_2_ gas was injected into the chamber through a needle valve and adjusted to a pressure of 90 Pa. The PDMS samples and capillary devices were then exposed for 30 s at 15 W to the plasma treatment. It is known that the PDMS polymer chain is composed of -OSi(CH_3_)_2_- repeating units. After the O_2_ plasma activation process, the methyl groups (CH_3_) are replaced by hydroxyl groups (-OH), forming silanol groups (Si-OH). The oxygen plasma oxidizes the surface, converting the native -OSi (CH_3_)_2_- form into the -O_n_Si (OH)_4-n_- form [29].

### 2.4. Blood Flow Experiments

#### 2.4.1. Microchannel Geometry and Fabrication

**Figure 5:**
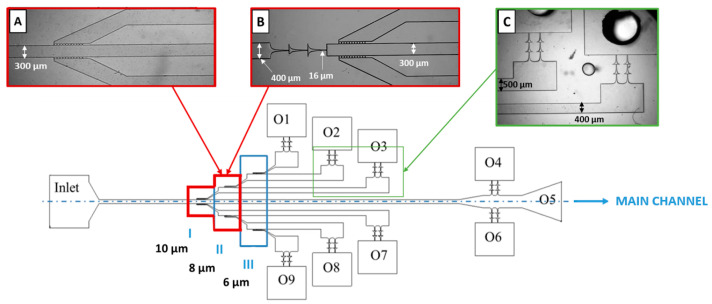
Schematic view of the microchannel design and its main dimensions. (**A**) Device 1, (**B**) Device 2, and (**C**) outlets (2.5× objective). Regions I, II, and III have cross-flow filtration barriers. The blue dashed line represents the axis of symmetry of the multi-step microfluidic device. Adapted from [30].

The geometric designs for the blood flow assays were developed based on the work of Faustino et al., 2022 [30] and designed in AutoCAD 2020 v23.1.47.0. Their molds were manufactured by the low-cost photolithography process described by Pinto et al., 2014 [31]. Two designed devices were employed, with major differences in the configuration of the main channel. In Device 1 (Figure 5A), the main channel features a straight rectangular channel with a width of 300 µm, whereas in Device 2 the main channel presents a double hyperbolic contraction (Figure 5B).

The microfluidic devices consisted of multiple stages of cross-flow filtration barriers (regions I, II, and III) and had a height of 30 µm. The main channel of Device 1 had a width of 300 µm while the branched channels were 400 µm and 500 µm wide, with a bifurcation of 45°, as represented in Figure 5A. The blood plasma separation takes place at various sequential pillar arrays, each made of ten rectangular pillars (50 × 55 µm) and with different gap sizes. Region I had a gap of 10 µm between pillars, whereas region II had a gap of 8 µm, and region III had a 6 µm gap size. The microchannel had nine outlets, eight of them preceded by two parallel sets of two hyperbolic contractions, as exhibited in Figure 5C. The hyperbolic contraction geometry had a 16 µm width at the smallest contraction size [30]. Device 2 differed only in the main channel, which presented a double hyperbolic contraction.

Devices with designs 1 and 2 were fabricated through replica molding from SU-8 molds [31] using control and bulk-modified PDMS. Only the bulk modification using 2.5% PEO was selected for this experiment. Firstly, liquid PDMS with ratio of 10:1 (*w*/*w*), control and bulk-modified, was poured into a Petri dish with the SU-8 molds. Following the thermal curing, the PDMS patterned with the microchannels was carefully cut and peeled off of the molds. Inlet and outlet holes were made by using a 1.5 mm biopsy punch. Subsequently, the PDMS surfaces were cleaned and safeguarded to prevent the accumulation of dirt or contaminants in the areas that would be sealed. The resulting microchannels were sealed by assembling the PDMS microchannels with the pre-cured PDMS layer (described in Section 2.3.2). The sealed microchannels were placed again in the oven for 30 min to further enhance and solidify the bonding.

#### 2.4.2. Blood Sample Processing Set-Up

Blood from healthy volunteer donors who provided informed consent was collected into 2.7 mL BD Vacutainer^®^ tubes. Samples (10 mL) containing 2% blood (*v*/*v*) diluted in physiological salt solution (PSS) (from Braun Medical, Melsungen, Germany) with 0.9% NaCl were prepared for the tests. The study was conducted in accordance with the Declaration of Helsinki and approved by the Hospital of Braga Ethics Committee.

**Figure 6:**
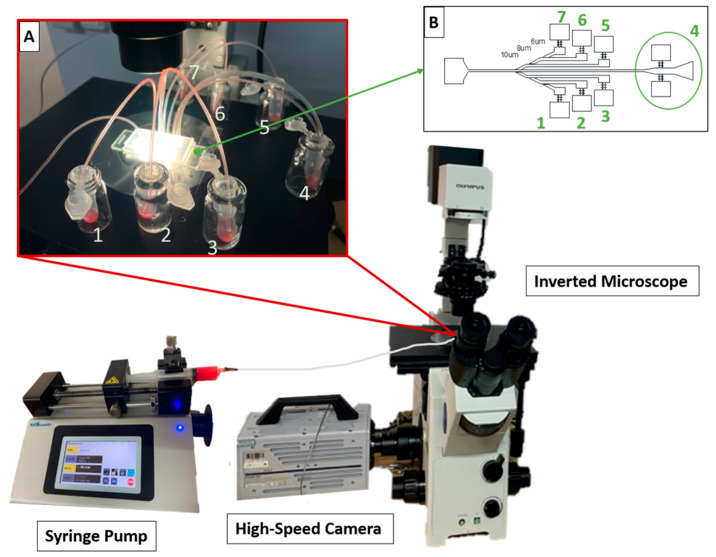
Schematic representation of the experimental set-up (inverted microscope coupled with a controllable syringe pump and a high-speed camera acquisition system). (**A**) Visual representation of sample collection system and (**B**) numbering of the outlets 1 to 7 (in green).

Each microchannel outlet was connected to an Eppendorf microtube (Sigma-Aldrich, Eppendorf®) for sample collection. For the blood flow studies, the experimental set-up included a high-speed microscopy video system consisting of an inverted microscope (IX71; Olympus Corporation, Tokyo, Japan) combined with a high-speed camera (Fastcam SA3, Photron, Motion Engineering Company, Westfield, IN, USA), as shown in Figure 6. Each PDMS device was placed and fixed on the microscope stage and image sequences were acquired. The flow rate of the working fluids was initially kept constant at 50 µL/min using a syringe pump (KD Scientific Inc., Holliston, MA, USA) with a 10 mL syringe (TERUMO, Leuven, Belgium). The first task was to stabilize the flow, ensuring that the diluted blood sample filled and circulated through all the channels at a constant velocity. After fluid flow stabilization, the flow rate was increased to 200 µL/min in order to improve the cell separation within the devices. After this point, the outlet samples started to be recovered. At the same time, images of the flow within the device were captured by the high-speed camera at a frame rate of 2000 frames/s, a shutter speed ratio of 1/40,000, and an image resolution of 1024 × 1024. Images in regions I, II, and II were obtained during the experiments. 

After the separation steps within the microfluidic device, the volume of the samples recovered in each microtube (Figure 6A) was qualitatively analyzed through image analysis. Images of each microtube were documented immediately after the experiments and after sample sedimentation (4 h). The images were transferred to the Image J 1.54d software and analyzed with the Plot Profile feature. Briefly, a plot profile of pixel intensity from a drawn line selection was obtained for each microtube, and the mean intensity was compared between images. The different concentrations of RBCs in the sample would result in different pixel intensities, i.e., a higher concentration corresponds to a lower intensity value. 

## 3. Results

### 3.1. Water Contact Angle (WCA) Measurements

**Figure 7:**
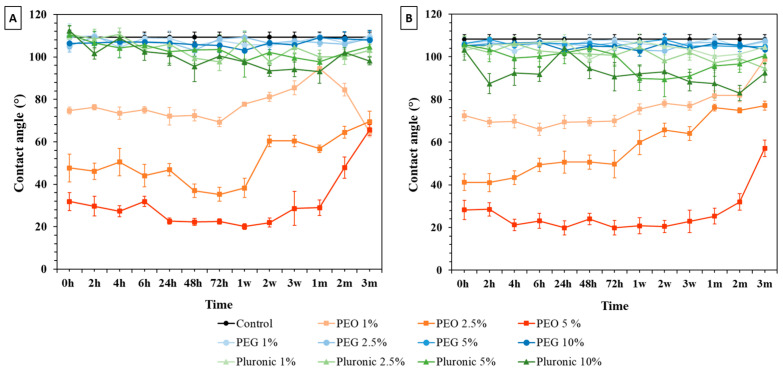
Water contact angle results from control and bulk-modified PDMS samples with (**A**) 10:1 ratio and (**B**) 5:1 ratio. Error bars represent the mean standard deviation at 95%, h –hours, w- weeks and m-months.

**Figure 8:**
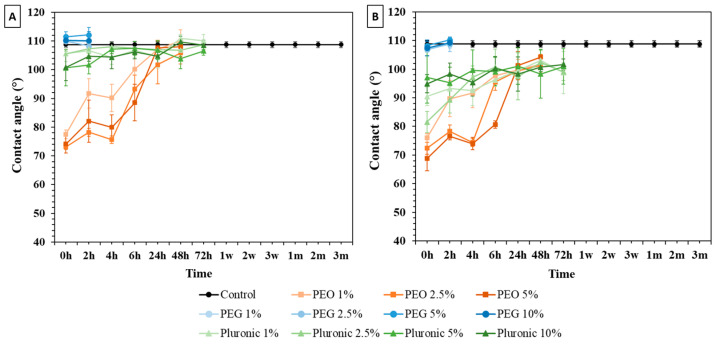
Water contact angle results from control and surface-immersion-modified PDMS samples: (**A**) 10:1 ratio and (**B**) 5:1 ratio. Error bars represent the mean standard deviation at 95%, h –hours, w- weeks and m-months.

The WCA between a droplet of water and the PDMS surface (control and modified) was measured. For the control PDMS samples at ratios of 10:1 and 5:1, the WCA was approximately 109.0 ± 2.4° (WCA ± SD) and 108.0 ± 2.1°, respectively (Figure 7 and Figure 8). This proves what has already been mentioned in the literature [10]: PDMS has a hydrophobic nature, with a WCA of approximately 110°.

#### 3.1.1. Bulk Modification 

The results of the bulk modification of PDMS at a ratio of 10:1 are represented graphically in Figure 7A. The WCA measurements showed wettability changes on the modified PDMS surfaces for all the tested percentages of PEO, Pluronic^®^ F127, and PEG. It was possible to observe an immediately reduction in the WCA of the modified samples in relation to the control PDMS samples at all time points. Regarding the PEG-modified samples, the WCA measurements did not reach below 100°, while the samples modified with Pluronic^®^ F127 had an WCA of around 80°. The WCA measurements for the modified PEO-PDMS samples exhibited a reduction in WCA to 22.5 ± 1.3° (t = 72 h) in the case of PEO 5%. For the 1 and 2.5% PEO samples, the WCA decreased to 69.3 ± 2.3° and 35.2 ± 3.3°, respectively. Studies on modified PDMS samples have shown that after a period of time, this elastomer recovers its hydrophobicity [29,32]. In the results obtained, a recovery of the hydrophobicity of the modified PDMS samples (i.e., CA increasing over time) was notable after only 1 week of treatment for the PEO-PDMS samples. 

The results of the bulk modification with PDMS at a 5:1 ratio are represented graphically in Figure 7B. The WCA measurements remained similar to those of the 10:1 PDMS samples. A reduction in WCA with an increase in surfactant percentage was observed. However, for the samples modified with PEG and Pluronic^®^ F127, the WCA measurements remained close to the control PDMS values and did not decrease below 80°. In general, for the 5:1 PEO samples, recovery was observed only after 1 month, and the lowest CA values were obtained for the 5% PEO samples. From the analysis of Figure 7, it is evident that the treatment that produced a more hydrophilic surface was the one using 5% PEO. PDMS chains are composed of silicon (Si) bonded to oxygen (O), forming siloxane (-Si-O-) bonds, whereas PEO chains consist of repeating ethylene oxide units (-CH_2_-CH_2_-O-). The PEO-modified PDMS incorporates PEO monomers into the PDMS matrix during polymerization, resulting in a more SiOx-rich layer and increasing the PDMS surface hydrophilicity. It is also important to note that, as the percentage of the surfactant increases, the WCA of the samples decreases, confirming the results obtained by Yao et al., 2012 [23].

#### 3.1.2. Surface Immersion Modification

The modified 10:1 PDMS samples with PEG and Pluronic^®^ F127 maintained WCA values close to those of the control, as shown in Figure 8A. However, in the treatments involving the addition of PEO, a reduction in the WCA was observed. The percentages of 1, 2.5, and 5% of PEO decreased the WCA of the modified PDMS to 77.5 ± 1.5°, 73.2 ± 0.9°, and 74.2 ± 3.2° (t = 0 h), respectively. However, a rapid recovery of hydrophobicity was observed after 4 h. The PEO-modified samples reached a WCA value of 100° within just 48 h. The measurements ended when the CA reached values close to that of the control samples.

The modified 5:1 PDMS samples (Figure 8B) have a WCA behavior close to the 10:1 samples. Examining the graph in Figure 8, it becomes evident that the most effective surfactant surface treatments are consistent with those for the 10:1 ratio samples, with the exception of the small decrease in the WCA values of the Pluronic F127 samples.

**Figure 9:**
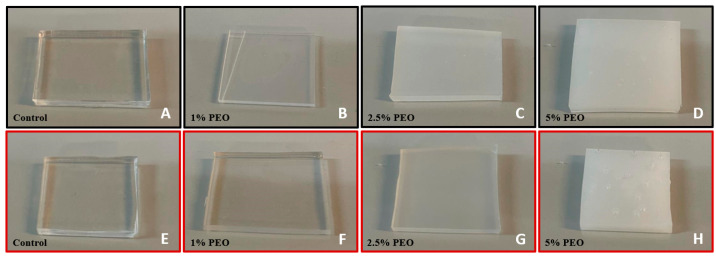
Comparison between the samples with ratios of 10:1 (black boxes, (**A**–**D**)) and 5:1 (red boxes, (**E**–**H**)). (**A**,**E**) are the control samples for each ratio. The PEO-modified samples are arranged from the highest to the lowest level of visibility.

In general, surface immersion modification is not as advantageous as bulk modification in the reduction of the surface’s WCA. It could, however, be advantageous when optical properties are to be considered. The optical property is of high importance when microscope visualizations within the devices are needed. The control samples (Figure 9A,E) were entirely translucent and served as a reference. In Figure 9, the varying degrees of whiteness of the PEO modified samples with the bulk procedure can be observed. It was visually possible to conclude that the samples treated with the highest percentage of surfactant had the poorest transparency. The highest level of transparency was observed in the 1% PEO samples with both ratios.

### 3.2. Capillary Flow Studies

**Figure 10:**
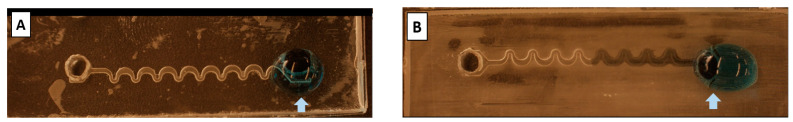
Spiral channels of 10:1 ratio control devices (**A**) without O_2_ plasma treatment and (**B**) with O_2_ plasma treatment, both with a 100 µL drop (light blue arrow).

Capillary flow tests were performed to evaluate the surface wettability by observing the self-movement of a fluid through the devices. A small volume of fluid (water, *v =* 100 μL) was pipetted onto the inlet, and its flow was monitored by measuring the time (in seconds) that it took to reach the device outlet. The PDMS microchannels were fabricated with control and bulk-modified PDMS. The outcomes for the control devices that were not subjected to the O_2_ plasma treatment demonstrated that PDMS’s hydrophobic nature inhibits the flow of a liquid, in this case, distilled water, through the channels (Figure 10). Subsequently, tests were conducted on the control devices treated with O_2_ plasma (Figure 10B). The rapid and immediate movement of the liquid was clearly observed.

Regarding the surfactant-modified devices, the bulk procedure with 2.5% PEO was used due to the beneficial WCA measurements and good transparency compared to higher concentrations of surfactant. The duration of the capillary flow for each microchannel was carefully measured and is presented in Table 1. Similar to the O_2_ plasma-treated microchannels, the flow started immediately as soon as the drop reached the inlet.

The results showed a significant increase in the capillary flow times for the channels subjected to the plasma treatment, indicating a recovery of the hydrophobic surface property. This suggests that this approach enhances capillary behavior, but only within an hour after activation. However, the devices modified with PEO exhibited insignificant changes in capillary flow, suggesting that the modification performed with the PEO surfactant at 2.5% could be a good candidate for PDMS surface modification for in vitro studies.

### 3.3. Blood Plasma Separation 

The blood flow studies were conducted on devices manufactured with control and modified PDMS with PEO 2.5%. Microscope images were captured during the flow visualizations, with a focus on the 5 min and 20 min time points of blood flow within the devices to observe the formation of agglomerates. Each device underwent testing using a total volume of 5 mL of a diluted blood sample (2% of total blood in PSS). The outlet volumes were collected across seven microtubes connected to each outlet to quantify the cell separation efficiency, as illustrated in Figure 6.

**Figure 11:**
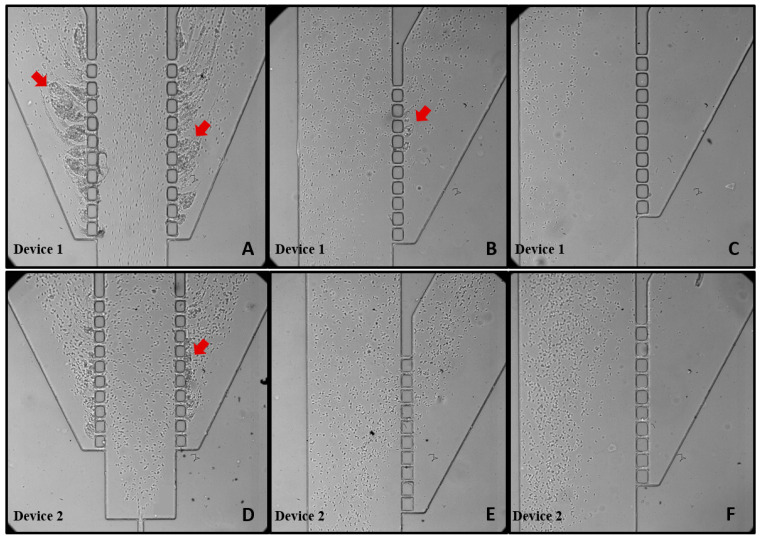
Images captured using a 10× objective lens plus a zoom of 1.6×. PDMS control Device 1: (**A**) zone I, (**B**) zone II, and (**C**) zone III; PDMS control Device 2: (**D**) zone I, (**E**) zone II, and (**F**) zone III. Clusters of cells are indicated with red arrows. All the representations are displayed at the 20 min mark with a flow rate of 200 µL/min.

In Device 1 (fabricated with control PDMS), after 5 min of testing, clusters of cells were already noticeable in regions I and II. By the 20 min mark, these clusters had grown in size, disrupting the flow dynamics within the device (Figure 11A–C). Upon concluding the test (after the 5 mL volume of the blood sample had passed through the device), the microtubes were gathered, and it was immediately evident that the complete separation of plasma from the blood had not been achieved. All samples presented a red color, showing that the RBCs were able to reach the respective outlet. Subsequently, images of the tubes containing the final samples were taken and analyzed using Image J.

The second test was conducted on Device 2, fabricated with control PDMS, which featured a double hyperbolic contraction geometry at the main channel. At the 5 min mark, clusters of cells were once again observed, and by the 20 min mark, these clusters had increased in size (Figure 11D–F). From Figure 11F, it is evident that in zone III, the flow guided the cells into the main channel, preventing most of them from passing through the pillars, demonstrating a partial blood plasma separation.

In microfluidic devices, the presence of a sudden expansion downstream of hyperbolic contractions alters the spatial distribution of cells and enhances the cell-free layer (CFL). In this context, Yaginuma et al., 2013 [33], and Rodrigues et al., 2015 [34], showed that microfluidic devices with a hyperbolic contraction followed by a sudden expansion (similar to the geometry of Device 2) can effectively separate RBCs from plasma. This corroborates our findings when comparing control Devices 1 and 2.

**Figure 12:**
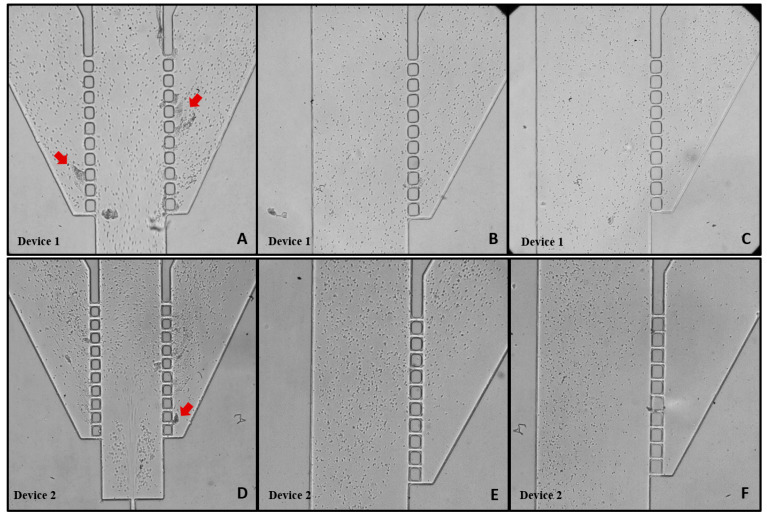
Images captured using a 10× objective lens plus a zoom of 1.6×. PEO Device 1: (**A**) zone I, (**B**) zone II, and (**C**) zone III; PEO Device 2: (**D**) zone I, (**E**) zone II, and (**F**) zone III. Clusters of cells are indicated with red arrows. All the representations are displayed at the 20 min mark with a flow rate of 200 µL/min.

The subsequent tests were conducted on similar devices, fabricated with modified PDMS through the bulk modification method with 2.5% PEO (Figure 12). The initial flow stabilization was promptly achieved and with an easy release of air bubbles. This rapid stabilization can be attributed to an enhanced flow caused by the hydrophilic walls of the microchannels.

After increasing the flow rate to 200 µL/min, Device 1 did not exhibit the formation of clusters at the 5 min mark. By the 20 min mark, small clusters had formed in region I only. Similar to Device 1, Device 2 displayed small clusters exclusively in region I, and the occurrence of a circular flow pattern downstream of the double hyperbolic contraction was observed (Figure 12D). Devices with hydrophilic walls are expected to decrease flow resistance, thereby increasing flow velocity. The formation of cell vortices downstream of the contraction indicates a higher velocity profile compared to devices with hydrophobic walls (Figure 12A). This observation aligns with those of Yao et al.’s 2012 study [23], which demonstrated that increasing the concentration of the surfactant (PEO) results in a faster fluid flow within the capillary channel. This distinctive cell recirculation was only observed in Device 2 modified with 2.5% PEO. This phenomenon could potentially be exploited for other applications, such as fluid mixing and cell trapping [35]. 

**Figure 13:**
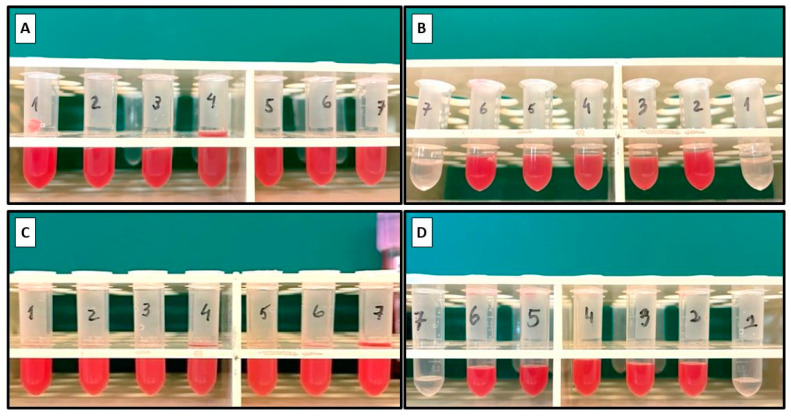
Captured images of the microtubes with the samples collected from each outlet of the devices (O1, O2, O3, O4, O5, O6, O7). Samples from (**A**) Device 1, control sample; (**B**) Device 2, control sample; (**C**) Device 1 modified with PEO 2.5%; and (**D**) Device 2 modified with PEO 2.5%.

Analyzing the microtubes containing the recovered samples from the respective outlets (Figure 13), it was observed that a total separation of plasma from blood had not occurred for control Devices 1 and 2. There was a more pronounced degree of separation in Device 2 (with the hyperbolic contraction) at outlets 1 and 7, which showed almost clear samples, i.e., with a small amount of RBCs (Figure 13B). By using the Image J plugin Plot Profile, it was possible to corroborate those findings. Outlets 1 and 7 of Device 2 (Figure 13B) present higher values of pixel intensity, i.e., clearer samples.

**Figure 14:**
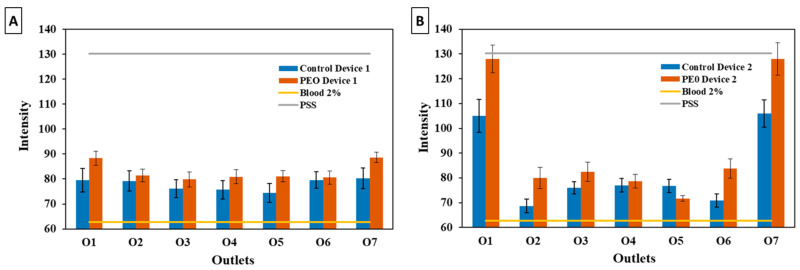
Representation of the mean pixel intensity value of the final samples from the device outlets obtained by the *Plot Profile* plugin for (**A**) Device 1 and (**B**) Device 2. The color intensity of the initial 2% blood sample (yellow line) and PSS (grey line) are also presented.

Figure 13C,D present the recovered outlet samples from the modified devices with 2.5% PEO. In the case of Device 1, the results were close to the ones obtained for the control device. For the modified Device 2, the results were better at outlets 1 and 7. Despite the smaller volume of sample recovered, no RBCs were observed, which was supported by the higher value of mean pixel intensity (Figure 14B). This was also proven by the proximity of the mean pixel intensity value to the PSS sample.

Figure 14A,B demonstrate that the final samples from the modified devices exhibited a lower pixel intensity of pigmentation in comparison to the control devices. This could be attributed to the improved flow characteristics, facilitated by the bulk surface modification. As a result, this led to a more efficient separation of plasma from total blood when compared to the control devices.

## 4. Conclusions

In this paper, the process of fabrication and surface wettability modification of PDMS samples for applications in microfluidic devices was developed and studied. The proposed modification procedures were performed in two distinct forms: in bulk and by surface immersion in a solution. Three surfactants, PEO, Pluronic^®^ F127, and PEG, were tested as the agents to enhance the hydrophilic behavior of the PDMS samples surface. It was possible to conclude that there was a more significant influence of the surfactants on PDMS wettability when bulk modifications were performed rather than when the immersion modification was performed. It is of some importance to highlight that, despite the existence of a decrease in the WCA of all samples with high percentage of surfactant, their transparency also decreased, i.e., they had lower optical visibility. After a careful analysis of the aforementioned treatments, it was the bulk modification method using 2.5% PEO that represented the most efficient surface modification. This choice was attributed to its favorable WCA values over time, optical transparency, and sustained hydrophilicity over an extended duration.

The difficulties experienced during the curing of the modified PDMS over the 3D printed molds were overcome by developing a PDMS double casting method to obtain PDMS microchannel molds. Using the PDMS molds, bulk-modified microdevices were fabricated for capillary flow studies. The PEO 2.5% bulk-modified devices exhibited favorable flows through the action of capillary forces, even 48 h after manufacturing.

Therefore, it is necessary to understand if the proposed PDMS surface modification is suitable for PDMS microfluidic device applications. Two proposed devices—one with a hyperbolic contraction (Device 2) and another without it (Device 1)— fabricated with the bulk-modified method were tested with healthy blood samples to test their ability to separate the blood cells from blood plasma. During the blood flow studies, it became evident that the geometry and wettability of the device exerted an influence on the separation of plasma from blood, and a significant improvement in the separation efficiency (almost pure plasma separation) with Device 2 was obtained. The introduction of PEO facilitated the blood flow experiments by reducing the presence of air bubbles and cell agglomerates and by facilitating the fluid flow. Thus, it can be concluded that the bulk modification performed with the PEO surfactant at 2.5% is a promising candidate for PDMS surface modification of microfluidic devices for in vitro blood studies such as those testing plasma separation. 

In summary, the optimized final conditions and insights from this work are as follows:

- Bulk surface modification emerged as the most efficient method for long-term PDMS surface wettability modification;
- The surfactant PEO at a concentration of 2.5% was identified as the most suitable option, taking into account the surface properties of PDMS such as the water contact angle (WCA), capillarity, and optical features;
- Device 2, featuring a double hyperbola-shaped contraction at the main channel branch, demonstrated superior efficiency for blood plasma separation compared to Device 1;
- The PEO bulk-modified Device 2 proved to be the most efficient microfluidic system for achieving pure plasma separation.

## Figures and Tables

**Table 1:** Capillary tests were conducted on the devices in Figure 3 at several time points. Measurements were obtained by measuring the fluid flow time (s).

Microchannels		Time (s)							
		Control PDMS		O_2_ Plasma Treatment			PEO 2.5%		
		0 h	2 h	0 h	2 h	48 h	0 h	2 h	48 h
**A**	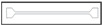	− *	− *	1.74	7.46	−*	7.80	8.10	9.20
**B**	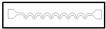	− *	− *	2.90	17.79	−*	27.27	25.15	26.30
**C**	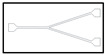	− *	− *	22.87	135.71	−*	6.02	6.30	6.10
**D**	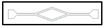	− *	− *	13.18	185.12	−*	5.48	5.88	5.54

* There was no movement of the fluid due to the hydrophobic nature of PDMS.

## Data Availability

The data supporting the conclusions of this article will be made available by the authors on request.
